# Oxidative Phosphorylation as a Predictive Biomarker of Oxaliplatin Response in Colorectal Cancer

**DOI:** 10.3390/biom14111359

**Published:** 2024-10-25

**Authors:** Toni Martinez-Bernabe, Daniel G. Pons, Jordi Oliver, Jorge Sastre-Serra

**Affiliations:** 1 Gruop Multidisciplinar de Oncología Traslacional, Institut Universitari d’Investigació en Ciències de la Salut (IUNICS), Universitat de les Illes Balears, 07122 Palma de Mallorca, Spain; toni.martinez@uib.es (T.M.-B.); d.pons@uib.es (D.G.P.); jorge.sastre@uib.es (J.S.-S.); 2 Instituto de Investigación Sanitaria de las Islas Baleares (IdISBa), Hospital Universitario Son Espases, Edificio S, 07120 Palma de Mallorca, Spain; 3 Ciber Fisiopatología Obesidad y Nutrición (CB06/03), Instituto Salud Carlos III, 28029 Madrid, Spain

**Keywords:** colorectal cancer, oxidative phosphorylation, biomarkers, oxaliplatin, resistance

## Abstract

Oxaliplatin is successfully used on advanced colorectal cancer to eradicate micro-metastasis, whereas its benefits in the early stages of colorectal cancer remains controversial since approximately 30% of patients experience unexpected relapses. Herein, we evaluate the efficacy of oxidative phosphorylation as a predictive biomarker of oxaliplatin response in colorectal cancer. We found that non-responding patients exhibit low oxidative phosphorylation activity, suggesting a poor prognosis. To reach this conclusion, we analyzed patient samples of individuals treated with oxaliplatin from the GSE83129 dataset, and a set of datasets validated using ROCplotter, selecting them based on their response to the drug. By analyzing multiple oxaliplatin-resistant and -sensitive cell lines, we identified oxidative phosphorylation KEGG pathways as a valuable predictive biomarker of oxaliplatin response with a high area under the curve (AUC = 0.843). Additionally, some oxidative phosphorylation-related biomarkers were validated in primary- and metastatic-derived tumorspheres, confirming the results obtained in silico. The low expression of these biomarkers is clinically relevant, indicating poor prognosis with decreased overall and relapse-free survival. This study proposes using oxidative phosphorylation-related protein expression levels as a predictor of responses to oxaliplatin-based treatments to prevent relapse and enable a more personalized therapy approach. Our results underscore the value of oxidative phosphorylation as a reliable marker for predicting the response to oxaliplatin treatment in colorectal cancer.

## 1. Introduction

Colorectal cancer (CRC) is the third most diagnosed cancer worldwide, becoming one of the biggest health problems globally. Chemotherapy is one of the common strategies among the different ways to deal with the disease [1]. Oxaliplatin is a chemotherapy drug commonly used for colorectal cancer treatment due to its efficacy in advanced stages of the tumor, when resection is no longer a viable option after the disease has spread [2]. On the other hand, while its benefits in the early stages of the tumor remain unclear [3,4,5]. Furthermore, oxaliplatin-based treatments present high recurrence rates (approximately 30%), becoming a significant barrier to the patient’s prognosis [6]. The efficacy and safety of oxaliplatin-based regimens in advanced-stage treatments have been inadequately reported. Some authors report that treatment with oxaliplatin in metastatic CRC patients showed a response rate (RR) of 21.6% (CI 14.3–31.0), with a higher response in patients who received oxaliplatin as adjuvant therapy (28.3%) compared to those treated in the metastatic setting (14.3%). Also, the disease control rate (DCR) was 57.8% [7]. Regarding safety, residual neuropathy was reported in 13% of patients after oxaliplatin retreatment, and grade 3 or higher adverse events were observed in approximately 25% of cases [7]. For this reason, the search for reliable markers to predict the response to treatment has become a necessary topic of study, not only to select the correct treatment for the patient, but also to avoid unnecessary side effects that worsen their quality of life.

Oxaliplatin is characterized by producing, among others, drug–DNA adducts that block DNA synthesis that impedes DNA replication and cellular division, although some cancer cells could become resistant to this treatment through different mechanisms [8,9]. Additionally, the response rates to oxaliplatin are still less than 60% [4], and oxaliplatin causes side effects resulting in dose-limiting toxicities [1]. Thus, research has emphasized the need for reliable biomarkers to predict the efficacy of oxaliplatin chemotherapy before treating patients to improve clinical outcomes. To our knowledge, different predictive oxaliplatin response markers have been described in colorectal cancer, such as oncogene mutations (KRAS) [10], excision repair protein levels (ERCC-1) [11], or microsatellite instability [12]. However, markers of efficacy for oxaliplatin have not, to date, achieved standard-of-practice usefulness. One of the models used to study chemotherapy resistance is the one using tumorspheres, which are based on the 3D culture of tumor cells [13], simulating the tumor structure and the heterogeneity in a more accurate way. The 3D cell culture heterogeneity arises from the cellular dedifferentiation that allows for the enrichment of cancer stem cells (CSCs) [14]. CSCs have been proposed as one of the possible causes of chemotherapy resistance in cancer, as well as being responsible for relapses [15].

Tumor-specific characteristics may reveal vulnerabilities that can serve as biomarkers. One of the most prominent tumor characteristics is tumor metabolism, which can adapt under low-nutrient conditions to increase tumor mass and maintain cell viability [16]. Metabolism plays a crucial role in treatment response, and some metabolites are promising biomarkers to predicting patient responses to treatment in different diseases [17]. One of the key regulators of metabolism are mitochondria, which are the energy engine of the cell, producing ATP through oxidative phosphorylation (OXPHOS) [18,19]. OXPHOS could be a key factor in treatment response due to its central role in metabolic adaptations in cancer cells, allowing them to obtain the necessary energy requirements [20] or even decrease its function to avoid higher cellular oxidative stress [21]. These metabolic adaptations demonstrate the relevant role that OXPHOS could have as a predictive factor in the response to treatment, as it has been observed in a CD8 T cell subset in immunotherapy-resistant melanoma patients [22].

To this day, it is unclear how OXPHOS expression is modulated in resistant colorectal tumors in response to oxaliplatin and whether OXPHOS could be used as a predictive marker for oxaliplatin response. For this reason, our aim is to analyze the predictive capacity of OXPHOS to determine if colorectal tumors would respond to oxaliplatin treatment. In this way, we intend to shed light on the research bases to approach new predictive markers of clinical outcomes.

## 2. Material and Methods

### 2.1. Reagents

Dulbecco’s modified Eagle’s medium High Glucose (DMEM), Fetal Bovine Serum (FBS), and antibiotics (penicillin and streptomycin) were purchased from BIOWEST (Riverside, MO, USA). The 3D Tumorsphere Medium, its Supplementation Mix, and DetachKit were purchased from PromoCell GmbH (Heidelberg, Germany). Oxaliplatin and Tri Reagent^®^ were purchased from Sigma-Aldrich (St. Louis, MO, USA). Routine chemicals were supplied by Bio-Rad Laboratories (Hercules, CA, USA), Sigma-Aldrich (St. Louis, MO, USA), and Panreac (Barcelona, Spain). The 6-well and 96-well ultra-low attachment (ULA) plates were purchased from SPL Life Sciences (Pocheon-si, Gyeonggi-do, Republic of Korea).

### 2.2. Collection of Data

The Affymetrix Human Gene 1.0 ST Array [transcript (gene) version] dataset (GSE83129) [23] was downloaded from the Gene Expression Omnibus data repository (GEO, https://www.ncbi.nlm.nih.gov/geo/, accessed on 22 February 2023). For the data analysis, two groups of patients were selected and compared according to their response to oxaliplatin (non-responders [n = 9] and responders [n = 17]). Differential gene expression analysis was performed using GEO2R. The *p*-value was adjusted for the correction of false-positive outputs when using the Benjamini and Hochberg (*FDR*: false discovery rate) method, and log_2_FC represents the fold changes in down- or upregulated genes. Further information is available in the original article [24].

### 2.3. GO, KEGG Pathway, and Over-Representation Enrichment Analysis

Differentially expressed genes (DEGs) collected from GEO after the comparison of non-responders vs responders patients grouped before were used to acquire the normalized enrichment scores (NES) of Gene Ontology: Cellular Component (GO–CC) and Kyoto Encyclopedia of Genes and Genomes (KEGG) pathways using a gene set enrichment analysis (GSEA) using the WEB-based GEne SeT AnaLysis Toolkit (WebGestalt). Only GO–CC terms and KEGG pathways with a *p*-value ≤ 0.05 and an *FDR* value ≤ 0.05 were considered significantly enriched, as previously described [25]. For the over-representation analysis (ORA), significant downregulated genes (log_2_(FC) < −0.5; *p*-value < 0.05) were selected and analyzed using WebGestalt.

### 2.4. Oxaliplatin Response Categorization

Analysis of oxaliplatin response categorization and ROC (receiver operator characteristic) analysis were performed by using the ROC plotter tool (https://www.rocplot.org, accessed on 25 December 2023). Half-maximal inhibitory concentration (IC50) and the area under the dose–response curve (AUDRC) values were considered to evaluate therapeutic response, as previously described by Fekete et al. [26]. Oxidative phosphorylation KEGG pathway (134 genes) expression was tested for oxaliplatin treatment in solid tumors. Using these genes, the system selects genes that significantly (*p* < 0.05) correlated to resistance using a Mann–Whitney test. Then, the significant genes were integrated using a random forest classifier into a single signature. Finally, a ROC analysis was used to evaluate the predictive effectiveness of this signature.

### 2.5. OXPHOS Gene Expression in Solid and Colorectal Tumors

Analysis of OXPHOS genes expression in solid and colorectal cancer tumors was performed by using the ROC plotter tool (https://www.rocplot.org, accessed on 25 December 2023). Data were collected from the GDSC2 dataset (https://www.cancerrxgene.org, accessed on 25 December 2023) and split into non-responder and responder groups based on lower and upper tertiles of IC50. A Mann–Whitney test was performed, and significance was set at *p* < 0.05.

### 2.6. Colon Tumorsphere Generation

Colorectal cancer cell lines belonging to the same patient obtained 1 year apart, SW480 primary tumor-derived cells (CCL-228), and SW620 metastatic cancer-derived cells (CCL-227) were purchased from American Type Culture Collection ATCC (Manassas, VA, USA) within the last 3 years and were maintained in DMEM supplemented with 10% FBS and 1% antibiotics (penicillin and streptomycin) at 37 °C with 5% CO_2_. Third-generation tumorspheres were obtained as previously described [27].

### 2.7. Sphere-Forming Efficiency (SFE) Assay

Secondary tumorspheres were dissociated, and 1.000 cells/well were seeded in 96-well ULA plates with vehicle or Oxaliplatin in 3D Tumorsphere Medium supplemented with Supplementation Mix for 96 h. Sphere-forming efficiency (SFE) was calculated as a percentage by dividing the tumorspheres formed per well by the number of cells seeded in the well, as previously described [27].

### 2.8. RT-qPCR

Secondary tumorsphere cells were dissociated and seeded in 6-well ULA plates with vehicle or Oxaliplatin in 3D Tumorsphere Medium supplemented with Supplementation Mix for 96 h to obtain the third-generation tumorspheres. RNA from cultured cells was isolated using Tri Reagent^®^, following the manufacturer’s protocol. Total RNA was quantified using a BioSpec-nano spectrophotometer (Shimadzu Biotech, Kyoto, Japan) set at 260 nm and 280 nm, obtaining a 260/280 nm ratio. cDNA was obtained using retrotranscription, and PCR reactions were carried out as previously reported [28]. Genes and their primers and annealing temperatures are shown in Supplementary Table S1. B2M and HMBS were used as housekeeping genes.

### 2.9. Cardiolipin Content and Mitochondrial Mass Analysis

Third generation tumorspheres were dissociated, and 1.5 × 10^4^ cells/well were seeded in 96-well plates with supplemented DMEM. After 24 h, cells were treated with vehicle (0.1% DMSO) or oxaliplatin 5 µM in 3D Tumorsphere Medium supplemented with Supplementation Mix for 96 h. Cardiolipin content was determined using fluorometric assays by staining cells with 250 nM of Nonyl Acridine Orange (NAO) to measure cardiolipin content, as previously described [29]. Mitochondrial mass was determined using fluorometric assays by staining cells with 10 nM Mitotracker Green (MTG, M7514, Invitrogen, Waltham, MA, USA) for 40 min at 37 °C and 5% CO_2_, as previously described [30]. The values obtained were normalized with the Hoechst 33342 fluorescence signal, as previously described [27].

### 2.10. Western Blot

Secondary tumorsphere cells were dissociated and seeded in 6-well ULA plates with vehicle or Oxaliplatin in 3D Tumorsphere Medium supplemented with Supplementation Mix for 96 h to obtain the third-generation tumorspheres. Then, tumorspheres were collected and lysed with RIPA buffer [28,31]. Protein concentration was determined using a bicinchoninic acid (BCA) protein assay kit from (Thermo Fisher Scientific, Waltham, MA, USA). Twenty micrograms of protein were resolved on a 15% SDS-PAGE gel and electrotransferred onto nitrocellulose membranes using the Trans-blot^®^ TurboTM transfer system (Bio-Rad, Hercules, CA, USA). Membranes were blocked in 5% non-fat powdered milk in TBS with 0.05% Tween for 1 h. The following were used as primary antibodies: antisera against OXPHOS cocktail (1:1000; ab110411, Abcam, Bristol, UK), COX4I1 (1:1000; ab33985, Abcam, Bristol, UK), and GAPDH (1:1000; sc-25778, Santa Cruz Biotechnology, Santa Cruz, CA, USA). Protein bands were visualized by using the ChemiDoc™ Imaging System (#12003153, Bio-Rad, Hercules, CA, USA) and following the protocol described by Sastre-Serra et al. [30].

### 2.11. Overall Survival and Relapse-Free Analysis

Overall survival (OS) (n = 1061) and relapse-free survival (RFS) (n = 1336) analyses of colorectal cancer patients were determined using the Kaplan–Meier plotter database (https://kmplot.com/analysis/, accessed on 25 December 2023). A LogRank *p*-value ≤ 0.05 was considered statistically significant.

### 2.12. Statistical Analysis

The statistical analyses were performed with the Statistical Program for the Social Sciences software for Windows (SPSS, version 27.0; SPSS Inc., Chicago, IL, USA). Data are presented as mean ± standard error of the mean (SEM). The Shapiro–Wilk test was performed to check whether variables followed a normal distribution. The statistical differences between vehicle- and oxaliplatin-treated cells were analyzed using a Student’s *t*-test or Mann–Whitney test, with statistical significance set at *p* < 0.05 (*).

## 3. Results

### 3.1. Characterization of Differentially Expressed Genes Between Oxaliplatin Non-Responder vs Responder Colorectal Cancer Patients

**Figure 1:**
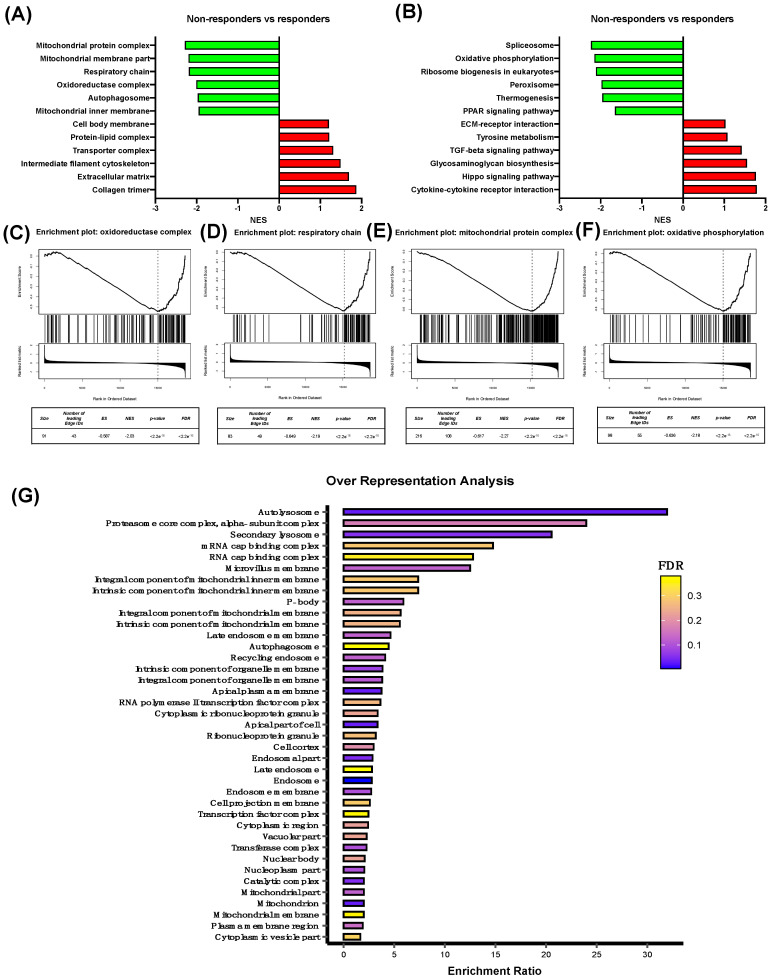
Characterization of differentially expressed genes between oxaliplatin non-responder and responder colorectal cancer patients. GO:CC (**A**) and KEGG (**B**) functional enrichment was performed using GSEA with identified DEGs in oxaliplatin non-responder colorectal patients compared to oxaliplatin responder colorectal patients from GSE83129 dataset. Green and red bars show the negative and positive NES values, respectively, of each GO:CC and KEGG pathway. Enrichment plots with their corresponding data (GO, size, number of leading edges, IDs, enrichment score, normalized enrichment score, *p*-value < 0.001, and *FDR* < 0.001) of oxidoreductase complex (**C**), respiratory chain (**D**), mitochondrial protein complex (**E**), and oxidative phosphorylation (**F**) are represented. An Over Representation Analysis (ORA) (**G**) was performed on all significant downregulated DEGs (log_2_(FC) < −0.5, *p* < 0.05).

Oxaliplatin non-responder and responder colorectal cancer (CRC) patients were selected from GSE83129 dataset, and differentially expressed genes (DEGs) were obtained from Gene Expression Omnibus (GEO). Gene set enrichment analysis (GSEA) software was used to analyze Kyoto Encyclopedia of Genes and Genomes (KEGG) pathway enrichment and the gene ontology cellular component (GO:CC) of all the DEGs. Non-responder patients showed a negative normalized enrichment score (NES), and therefore a downregulation expression of the related genes, in GO:CC categories (*p*-value < 0.05, *FDR* < 0.05) such as the mitochondrial protein complex, mitochondrial membrane part, respiratory chain, oxidoreductase complex, autophagosome, and mitochondrial inner membrane compared to oxaliplatin responder patients (Figure 1A). Additionally, some of the GO:CC terms that showed positive NESs were the cell body membrane, protein–lipid complex, transporter complex, intermediate filament cytoskeleton, extracellular matrix, and collagen trimer. To further examine the cellular processes implicated in GO:CC regulation, we observed that non-responder CRC patients presented some related KEGG pathways with negative NESs (*p*-value < 0.05, *FDR* < 0.05), such as spliceosome, oxidative phosphorylation, ribosome biogenesis in eukaryotes, peroxisome, thermogenesis, and the PPAR signaling pathway (Figure 1B). Regarding the KEGGs pathways with a positive NES, we observed ECM–receptor interactions, tyrosine metabolism, the TGF-beta signaling pathway, glycosaminoglycan biosynthesis, the Hippo signaling pathway, and cytokine–cytokine receptor interactions. To complete our aim, we delved deeper into the mitochondrial-related GO:CC and KEGG pathways, observing that oxaliplatin non-responder patients showed significant downregulation (*p*-value < 0.001, *FDR* < 0.001) of the oxidoreductase complex, respiratory chain, and mitochondrial protein complex (Figure 1C–E), as well as oxidative phosphorylation (Figure 1F). Moreover, an over-representation analysis of all significant downregulated genes (log_2_(FC) < −0.5, *p*-value < 0.05) was performed using WebGestalt, and, interestingly, mitochondrion (GO:CC) showed a statistically significant *FDR* (*p*-value < 0.001, *FDR* < 0.001) (Figure 1G).

### 3.2. Analysis of Oxidative Phosphorylation as a Predictor of Response to Oxaliplatin in Solid Tumors

**Figure 2:**
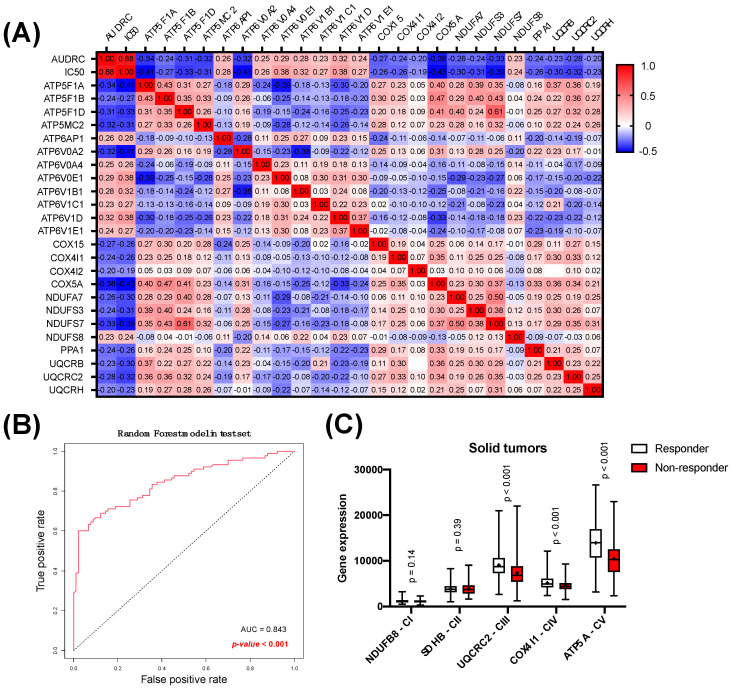
Oxidative phosphorylation as a predictor marker of oxaliplatin non-responder solid tumors. Correlation between oxidative phosphorylation genes related to resistance against oxaliplatin. A correlation matrix between drug screening results (AUDRC and IC50) and gene expressions using the GDSC2 dataset is shown (**A**). The chart includes only significant genes of the KEGG oxidative phosphorylation pathway, for which the Spearman correlation coefficient (when comparing IC50 to AUDRC) was below −0.20 or over 0.20. ROC curve of the random forest model analysis performed on the most significant genes correlated with oxaliplatin resistance (responder: n = 69; non-responder n = 21) (**B**). OXPHOS gene expression in oxaliplatin non-responder and responder colorectal tumor-derived cell lines. A Mann–Whitney test was performed to analyzed OXPHOS-related gene expression in oxaliplatin responder and non-responder solid tumors. Significance was set at *p* < 0.05 (responder: n = 113; non-responder: n = 114) (**C**).

From the 134 genes included in the oxidative phosphorylation pathway, 57 showed significant association with resistance to oxaliplatin treatment in the GDSC2 dataset (Supplementary Table S2). A correlation matrix between drug screening results (AUDRC—Area Under the Drug-Response Curve—and IC50) and gene expression revealed the influence of individual genes on each other (Figure 2A). Only significant genes, for which the Spearman correlation coefficient was below −0.20 or over 0.20 when comparing their expression to IC50 or AUDRC in the GDSC2 dataset, were shown. Also, the correlation between each gene is shown, resulting in a matrix of twenty-four 26 × 26 oxidative phosphorylation pathway-related genes, plus IC50 and AUDRC correlations. In this analysis, we showed that different subunits of the various complexes of the mitochondrial respiratory chain (complex I, III, IV, and V) were downregulated in oxaliplatin non-responder solid tumor-derived cell lines. However, some complex V subunits were upregulated (ATP6AP1, ATP6VOA4, ATP6V1B1, ATP6V1C1, ATP6V1D, and ATP6V1E1), as well as one complex I subunit (NDUFS8). To determine if OXPHOS-related genes could predict the response to an oxaliplatin random forest analysis on significant OXPHOS oxaliplatin resistance-related genes, afterward, a Mann–Whitney test was performed. ROC analysis was displayed after the clustering, showing statistically significant ROC AUC values (AUC = 0.843, *p* < 0.001) (accuracy = 0.756, sensitivity = 0.767, specificity = 0.744, precision = 0.75) (Figure 2B). Gene expression of some OXPHOS-related markers (NDUFB8, SDHB, UQCRC2, COX4I1, and ATP5A) was analyzed individually, showing statistically significant downregulation of UQCRC2 (complex III), COX4I1 (complex IV), and ATP5A (complex V) in oxaliplatin non-responder solid tumors (Figure 2C).

### 3.3. Validation of Oxidative Phosphorylation as a Predictor of Response to Oxaliplatin in Colorectal Tumorspheres

**Figure 3:**
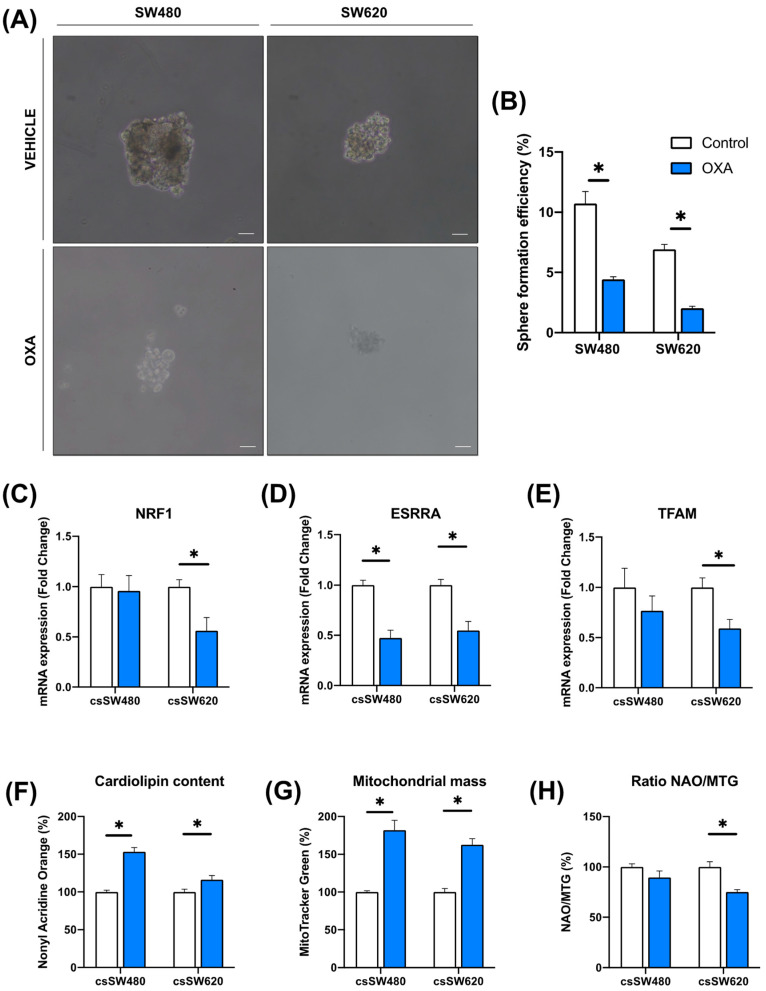
Evaluation of the expression of upstream markers of oxidative phosphorylation and mitochondrial differentiation in colorectal tumorspheres as a model of drug resistance. Representative images of third-generation colorectal tumorspheres derived from SW480 and SW620 cell lines (csSW480 and csSW620, respectively) in a vehicle- and oxaliplatin-treated condition are shown (**A**). Sphere formation efficiency (SFE) was analyzed after oxaliplatin treatment in SW480- and SW620-derived tumorspheres (**B**). mRNA expression of NRF1 (**C**), ESRRA (**D**), and TFAM (**E**) upstream OXPHOS-related markers were determined in SW480- and SW620-derived tumorspheres after oxaliplatin treatment, as well as the cardiolipin content (**F**), mitochondrial mass (**G**), and mitochondrial differentiation (**H**). Student’s *t*-test or Mann–Whitney test were performed to determine the significance between the experimental groups. Statistical significance was set at * *p* < 0.05 (n ≥ 4).

Using tumorsphere culture is a well-known method for generating drug resistance models [13]. Thus, we generated third-generation colorectal tumorspheres to check if oxidative phosphorylation is downregulated in a drug resistance model in response to oxaliplatin. Firstly, we observed that sphere formation efficiency was downregulated in both colorectal cancer cell lines (Figure 3A,B). Upstream OXPHOS gene regulators were analyzed in SW480 and SW620 cell lines, observing a decrease in NRF1, ESRRA, and TFAM expression in the metastatic SW620 cell line after oxaliplatin treatment, while only ESRRA downregulation was observed in the primary SW480 cell line (Figure 3C–E). Additionally, both SW480 and SW620 cell lines showed an increase in cardiolipin content and mitochondrial mass after oxaliplatin treatment (Figure 3F,G). However, only the SW620 cell line showed less mitochondrial differentiation, calculated using the NAO/MTG ratio (an indicator of mitochondrial cristae/mitochondrion), in response to oxaliplatin (Figure 3H).

**Figure 4:**
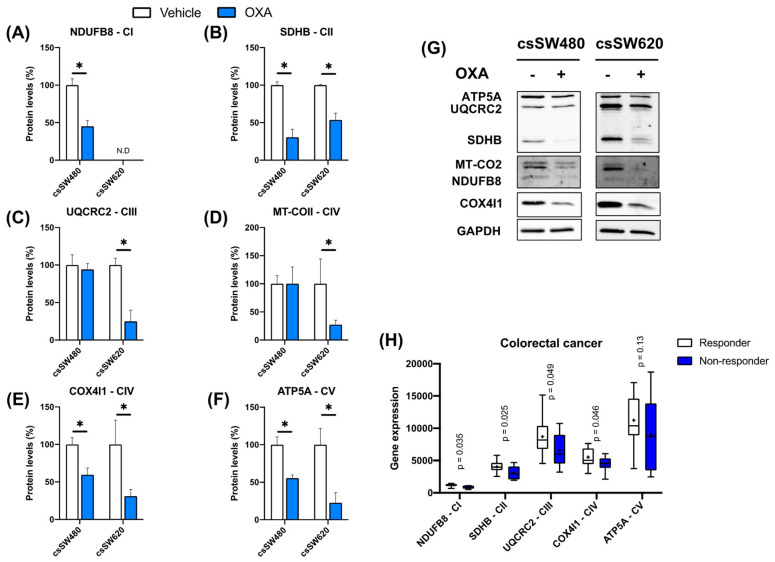
Validation of the OXPHOS protein expressions in colorectal tumorspheres after oxaliplatin treatment. NDUFB8 (**A**), SDHB (**B**), UQCRC2 (**C**), MT-COII (**D**), COX4I1 (**E**), and ATP5A (**F**) protein expression analyzed using Western blotting in third-generation colorectal tumorspheres derived from SW480 and SW620 cell lines in a vehicle- and oxaliplatin-treated condition, and their respective bands (**G**). Student’s *t*-test or Mann–Whitney test were performed to determine the significance between the experimental groups. Statistical significance was set at * *p* < 0.05 (n ≥ 4) (N.D: not detected). OXPHOS gene expression in oxaliplatin non-responder and responder colorectal tumors (**H**). A Mann–Whitney test was performed, and significance was set at *p* < 0.05 (responder: n = 14; non-responder: n = 4). Western blot original images are in the Supplementary Materials.

OXPHOS protein expressions were evaluated in colorectal tumorspheres in response to oxaliplatin, as well as in oxaliplatin non-responder and responder colorectal tumor-derived cell lines from GDSC2 dataset. Oxaliplatin significantly decreased NDUFB8, SDHB, COX4I1, and ATP5A protein levels in the SW480 cell line. On the other hand, SW620 showed a strong decrease in SDHB, UQCRC2, MT-COII, COX4I1, and ATP5A protein levels, while NDUFB8 protein levels were undetectable (Figure 4A–G). Furthermore, NDUFB8, SDHB, UQCRC2, and COX4I1 gene expression showed a statistically significant decrease in non-responder CRC tumor-derived cell lines after oxaliplatin treatment, while no significant effects were observed in ATP5A gene expression (Figure 4H).

### 3.4. Clinical Significance of Low OXPHOS Expression in Colorectal Cancer

**Figure 5:**
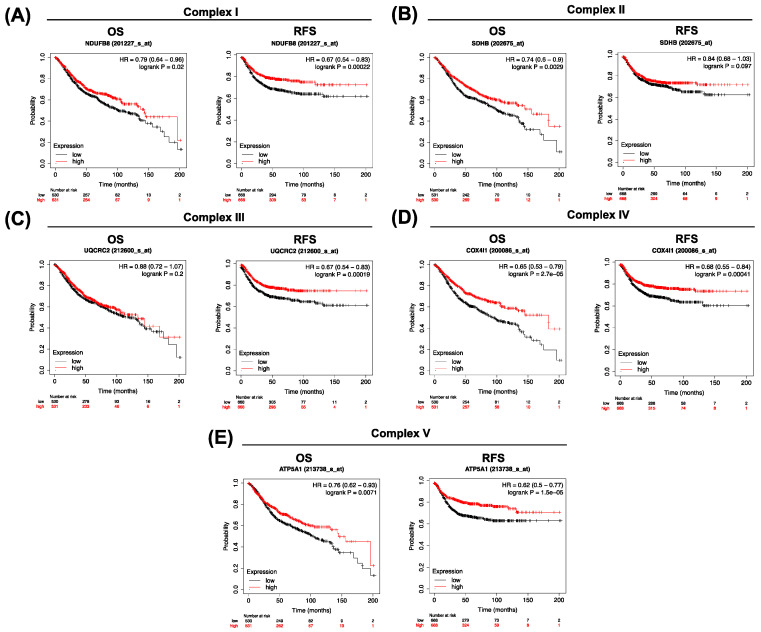
Clinical significance of OXPHOS expression in colorectal cancer. Overall survival and relapse-free survival analysis of colorectal cancer patients. Kaplan–Meier survival analysis comparing high (red) and low (black) mRNA expression of NDUFB8 (**A**), SDHB (**B**), UQCRC2 (**C**), COX4I1 (**D**), and ATP5A (**E**).

To determine the clinical significance of low OXPHOS gene expression in colorectal cancer patients’ outcomes, KMplots of overall survival (OS) and relapse-free survival (RFS) were performed. Low gene expression of NDUFB8 (201227_s_at), SDHB (202675_at), COX4I1 (200086_s_at), and ATP5A (213738_s_at) is related to worse overall survival (Figure 5A–E), while low gene expression of NDUFB8, UQCRC2 (212600_s_at), COX4I1, and ATP5A is related to worse relapse-free survival (Figure 5A–E).

## 4. Discussion

Oxaliplatin is commonly used for colorectal cancer treatment due to its efficacy in advanced stages of the tumor. However, not only its benefits remain unclear in the early stages of the tumor, but oxaliplatin-based treatments also present high recurrence rates (approximately 30%) [6]. For this reason, the aim to find predictive markers of the response to oxaliplatin becomes more relevant and necessary. In this study, a search for predictive biomarkers of response to oxaliplatin was conducted in a cohort of patients with oxaliplatin-sensitive and -resistant colorectal cancer. The results were validated in an in vitro chemoresistance model based on the culture of tumorspheres and were revalidated with different cohorts of oxaliplatin-resistant colorectal cancer. The colorectal cancer cell lines used for the formation of the tumorspheres were isolated from the same patient one year apart, with one originating from a primary tumor and the other from a metastatic tumor. This study analyzes the predictive capacity of oxidative phosphorylation to determine if colorectal cancer would respond to oxaliplatin treatment CRC (SW480), and the other from a metastatic-derived CRC (SW620).

Over the last few decades, much cancer research has focused on the search for predictive markers of response to anticancer treatments. In our study, we clearly found the ways in which mitochondria play a relevant role in the response to oxaliplatin. In fact, we observed that non-responder patients presented a downregulation of various mitochondrial components, such as the respiratory chain, mitochondrial protein complex, and oxidoreductase complex, among others (negative NES). These downregulations correlate with the diminution in the oxidative phosphorylation KEGG pathway in oxaliplatin non-responder patients. Mitochondria are dynamic organelles that modulate the metabolism in different conditions to meet requirements [32,33]. This dynamic behavior is one of the reasons that make mitochondria potential targets for cancer treatment, as they are capable of conferring drug resistance in tumor cells [34]. Our results prove that oxidative phosphorylation is downregulated not only in oxaliplatin non-responder patients, but also in oxaliplatin-resistant cell lines derived from all solid tumors and, more specifically, colorectal tumors. Indeed, as shown in the random forest model applied to the ROC curve analysis, highly activated oxidative phosphorylation could be a predictive model of oxaliplatin response. Moreover, the correlation matrix of drug screening results showed various significant OXPHOS-related genes that are mostly downregulated in oxaliplatin non-responder cell lines, such as ATP5A, COX4I1, or UQCRC2.

Colorectal cancer is well-known for its heterogeneous cell population along the crypts [35]. Drug administration could affect cellular diversity within the tumor differently, in which cancer stem cells (CSCs) are present [36]. These cells with low proliferation rates and high self-renewable capacities are considered to be responsible for chemotherapy resistance [15]. Furthermore, CSCs could downregulate OXPHOS to avoid excessive reactive oxygen species production in the mitochondrial respiratory chain and remain in a quiescent state, thus evading the damage caused by chemotherapy [37]. Thus, our findings in colorectal patients and drug-tested cell line databases were validated in primary- and metastatic-derived colorectal tumorspheres, which are considered to be a model of chemoresistance with an enriched population of CSCs [13]. The remaining resistant cell population after oxaliplatin treatment showed higher levels of cardiolipin and mitochondria, probably to counteract the strong decrease in OXPHOS protein levels, such as NDUFB8, SDHB, COX4I1, and ATP5A, in both primary and metastatic-derived tumorspheres. Interestingly, UQCRC2 and MT-COII protein levels only decreased in SW620-derived tumorspheres, which also showed less mitochondrial differentiation. These data highly correlate with OXPHOS gene expression in oxaliplatin non-responder solid and colorectal cancer cell lines, but also with the OXPHOS downregulation observed in non-responder patients. In addition, SW620-derived tumorspheres presented a decrease in upstream OXPHOS genes such as NRF1, ESRRA, and TFAM, while surprisingly, SW480-derived tumorspheres only decreased ESRRA gene expression after oxaliplatin treatment. The different effect on upstream OXPHOS gene expression could be due to the intrinsic differences between both cell lines as one become from a primary tumor and the other from a metastatic site. Also, this could explain how SW620-derived tumorspheres downregulated MT-COII levels, unlike SW480-derived tumorspheres.

In the literature, there is some controversy about the role of OXPHOS in resistance to certain drugs, since some authors suggest that OXPHOS increases its activity, while others indicate that OXPHOS decreases its activity due to the Warburg effect [38,39,40]. However, some previous studies were based on 2D cell culture models, where the focus may be different due to the high dynamic regulation of mitochondria, which are differently modulated depending on the conditions. For this reason, this study validates the results obtained from different databases in a 3D cell culture model. It is noteworthy that it has been described that resistant tumors decrease OXPHOS levels to reduce stress conditions under chemotherapy treatment [41]. In fact, we observed from clinical data that colorectal cancer patients have lower overall survival when the expressions of the NDUFB8, SDHB, COX4I1, and ATP5A genes are low in the tumor, which correspond with the OXPHOS protein levels of the remaining resistant population after oxaliplatin treatment. Furthermore, NDUFB8, UQCRC2, COX4I1, and ATP5A low levels were associated with lower relapse-free survival in colorectal cancer patients.

This study validates the expression of OXPHOS genes as a predictive biomarker for oxaliplatin response in colorectal cancer patients. These findings are potentially applicable in clinical settings, as patient-derived organoids are already being tested as a reliable model to predict patient response to different drugs. Measuring OXPHOS expression could be an accessible and rapid method for predicting responses to oxaliplatin treatment, thereby improving treatment criteria for patients. However, this study presents some limitations, such as the need for a larger sample size to avoid bias, as well as for further studies incorporating in vivo models and patient-derived xenografts.

## 5. Conclusions

This study analyzes the downregulation of mitochondrial cellular components, such as oxidative phosphorylation proteins, in colorectal cancer under oxaliplatin-resistant conditions. These findings were observed in models of colorectal cancer: in patients, in oxaliplatin-resistant cell lines, and in a validated drug-resistant tumorsphere model. Therefore, this study proposes using oxidative phosphorylation-related protein expression levels as a predictor of responses to oxaliplatin-based treatments to prevent relapse and enable a more personalized therapy approach. Our results underscore the value of oxidative phosphorylation as a reliable marker for predicting the response to oxaliplatin treatment in colorectal cancer.

## Data Availability

All data needed to evaluate the conclusions in the paper are included in the paper and Supplementary Materials. Additional data related to this paper are available from the corresponding authors upon request.

## Supplementary Materials

The following supporting information can be downloaded at: https://www.mdpi.com/article/10.3390/biom14111359/s1, Table S1: Primers and conditions used for RT-qPCR; Table S2: Significant oxidative phosphorylation-related DEGs associated with oxaliplatin resistance in solid tumors; Western blot original images.
