# Supplementary material for: Oxidative Phosphorylation as a Predictive Biomarker of Oxaliplatin Response in Colorectal Cancer
Source: Biomolecules. 2024 Oct 25;14(11):1359. doi: 10.3390/biom14111359 (PMC11591675; doi:10.3390/biom14111359)
Supplement: Supplementary file 1 [file biomolecules-14-01359-s001.zip › Supplementary Table S1.pdf]

Supplementary Table S1: Primers and conditions used for RT-qPCR

Gene<br>Accession number	Forward Primer (5'-3')<br>Reverse Primer (5'-3')
B2M	TTTCATCCATCCGACATTGA
NM_004048	CGGCAGGCATACTCATCTTT
ESRRA	TCGCTCCTCCTCTCATCATT
NM_004451.5	TGGCCAAACCCAAAAATAAA
HMBS	GAGAAGTCCAAGCAACAGC
NM_001024382.2	CCTTCAGAACTGGTTTATTAGTAGG
NRF1	CCACGTTACAGGGAGGTGAG
NM_005011.5	TGTAGCTCCCTGCTGCATCT
TFAM	GTGGTTTTCATCTGTCTTGGC
NM_003201.3	ACTCCGCCCTATAAGCATCTT
