# Supplementary material for: Oxidative Phosphorylation as a Predictive Biomarker of Oxaliplatin Response in Colorectal Cancer
Source: Biomolecules. 2024 Oct 25;14(11):1359. doi: 10.3390/biom14111359 (PMC11591675; doi:10.3390/biom14111359)
Supplement: Supplementary file 1 [file biomolecules-14-01359-s001.zip › Supplementary Table S2.pdf]

Gene	Fold Change	Mann-Whitney p-value	AUC	ROC p-value	FDR (p-adjusted)
COX5A	0.771	3.0e-21	0.737	0.0e+00	3.0e-19
ATP5F1A	0.748	5.2e-20	0.729	0.0e+00	2.6e-18
NDUFS7	0.763	2.0e-19	0.726	0.0e+00	6.5e-18
ATP6V0E1	1.4	1.5e-17	0.714	0.0e+00	3.7e-16
ATP6V0A2	0.662	4.1e-17	0.711	0.0e+00	8.2e-16
ATP6V1D	1.36	2.0e-16	0.706	0.0e+00	3.2e-15
ATP5MC2	0.818	8.3e-15	0.695	0.0e+00	1.2e-13
ATP5F1D	0.733	3.5e-14	0.69	0.0e+00	4.4e-13
UQCRC2	0.814	5.0e-14	0.689	1.1e-16	5.5e-13
NDUFA7	0.828	7.3e-12	0.672	8.3e-14	7.2e-11
ATP6AP1	1.33	1.2e-11	0.67	1.5e-13	1.1e-10
ATP6V1B1	1.84	1.9e-11	0.668	2.4e-13	1.5e-10
ATP5F1B	0.882	2.8e-11	0.667	5.9e-13	2.2e-10
UQCRH	0.762	5.0e-11	0.665	8.7e-13	3.5e-10
UQCRB	0.805	2.3e-10	0.659	9.1e-12	1.5e-09
ATP6V0A4	0.796	3.0e-10	0.658	1.3e-11	1.7e-09
ATP6V1E1	1.24	2.8e-10	0.658	8.2e-12	1.7e-09
COX15	0.854	3.8e-10	0.657	1.8e-11	2.1e-09
ATP6V1C1	1.25	5.0e-10	0.656	2.1e-11	2.5e-09
NDUFS3	0.894	4.5e-10	0.656	1.7e-11	2.3e-09
PPA1	0.858	1.7e-09	0.651	1.3e-10	7.9e-09
COX10	0.89	2.0e-09	0.65	9.5e-11	8.9e-09
COX4I1	0.89	7.4e-08	0.635	1.1e-08	3.2e-07
ATP5ME	0.881	4.0e-07	0.627	8.2e-08	1.6e-06
ATP5PB	0.906	3.9e-07	0.627	6.8e-08	1.6e-06
ATP5MC3	0.915	6.5e-07	0.625	1.6e-07	2.5e-06
NDUFS8	1.12	7.9e-07	0.624	1.4e-07	2.9e-06
COX7A2L	0.854	2.9e-06	0.617	6.4e-07	1.0e-05
COX6C	0.957	7.1e-06	0.613	2.5e-06	2.3e-05
NDUFAB1	0.898	7.1e-06	0.613	1.9e-06	2.3e-05
ATP6V1B2	0.846	3.9e-05	0.603	1.2e-05	1.3e-04
COX7B2	1.65	6.7e-05	0.6	2.2e-05	2.1e-04
COX4I2	0.935	8.8e-05	0.598	3.1e-05	2.6e-04
ATP6V0D1	1.18	1.0e-04	0.597	3.5e-05	2.9e-04
`MT-ND4`	1.07	1.7e-04	0.594	6.2e-05	4.7e-04
ATP5MG	0.853	5.9e-04	0.586	2.4e-04	1.6e-03
ATP5PF	0.928	8.8e-04	0.583	3.8e-04	2.4e-03
COX11	0.908	9.8e-04	0.583	4.1e-04	2.6e-03
UQCRQ	1.07	1.1e-03	0.582	4.7e-04	2.7e-03

ATP6V1A	1.12	1.5e-03	0.58	6.5e-04	3.7e-03
ATP5F1C	0.95	1.5e-03	0.579	7.0e-04	3.7e-03
`MT-CO3`	1.03	2.4e-03	0.576	1.1e-03	5.6e-03
ATP6V1G3	0.995	2.6e-03	0.575	1.2e-03	5.9e-03
NDUFV1	0.963	2.6e-03	0.575	1.2e-03	5.9e-03
COX6B1	0.954	3.1e-03	0.574	1.5e-03	6.8e-03
COX7A2	0.949	3.5e-03	0.573	1.6e-03	7.3e-03
NDUFS6	1.12	3.4e-03	0.573	1.7e-03	7.2e-03
NDUFS2	0.936	5.1e-03	0.57	2.3e-03	1.0e-02
NDUFV3	1.11	7.3e-03	0.567	3.4e-03	1.5e-02
PPA2	0.94	8.5e-03	0.566	4.0e-03	1.7e-02
ATP6V1E2	0.943	1.4e-02	0.561	7.0e-03	2.8e-02
ATP5F1E	1.06	1.6e-02	0.56	7.8e-03	3.1e-02
ATP6V0A1	0.918	2.8e-02	0.555	1.4e-02	5.1e-02
ATP6V0D2	1.11	2.7e-02	0.555	1.3e-02	5.1e-02
NDUFB5	0.935	3.9e-02	0.552	1.9e-02	7.1e-02
NDUFB2	1.07	4.3e-02	0.551	2.1e-02	7.7e-02
ATP6V0B	0.931	4.6e-02	0.55	2.3e-02	8.0e-02

Supplementary Table S2: Significant oxidative phosphorylation-related DEGs associated with oxaliplatin resistance in solid tumors.
