# Supplementary material for: Oxidative Phosphorylation as a Predictive Biomarker of Oxaliplatin Response in Colorectal Cancer
Source: Biomolecules. 2024 Oct 25;14(11):1359. doi: 10.3390/biom14111359 (PMC11591675; doi:10.3390/biom14111359)
Supplement: Supplementary file 1 [file biomolecules-14-01359-s001.zip › uncropped WB.pdf]

## <u>Western blot</u>

***csSW480***

C OXA C OXA C OXA

ATP5A -54 kDa
UQCRC2 – 48 kDa

SDHB – 29 kDa

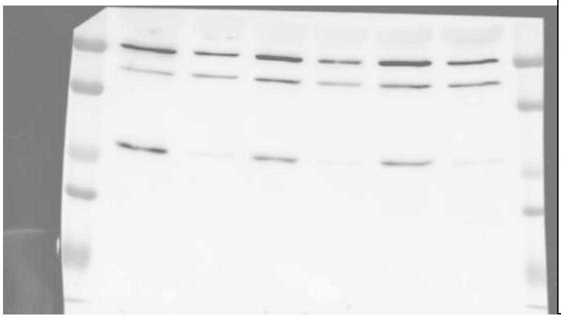

Mk (KDa)
50
37

25
20

15

C OXA C OXA C OXA

MT-COII – 22 kDa
NDUFB8 – 18 kDa

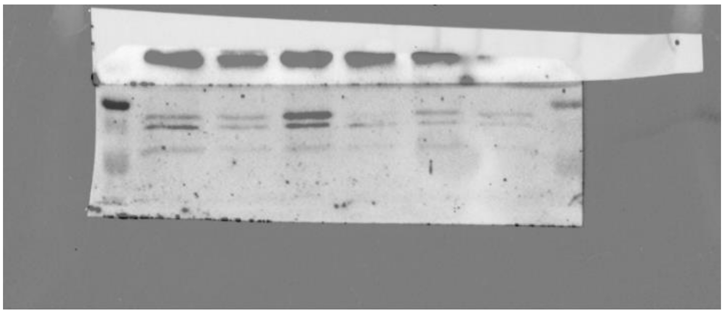

Mk (KDa)
20

15

C OXA C OXA C OXA

COXIV – 17 kDa

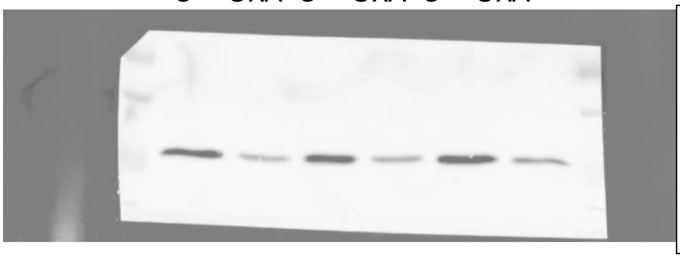

Mk (KDa)
25
20

15

C OXA C OXA C OXA

GAPDH – 37 kDa

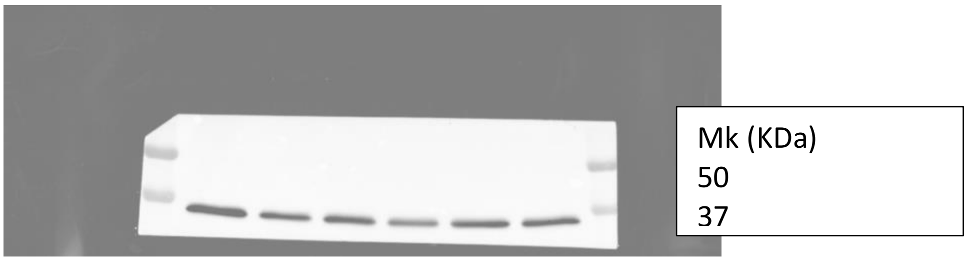

Mk (KDa)
50
37

***csSW620***

X OXA C OXA C OXA

ATP5A -54 kDa
UQCRC2 – 48 kDa

SDHB – 29 kDa

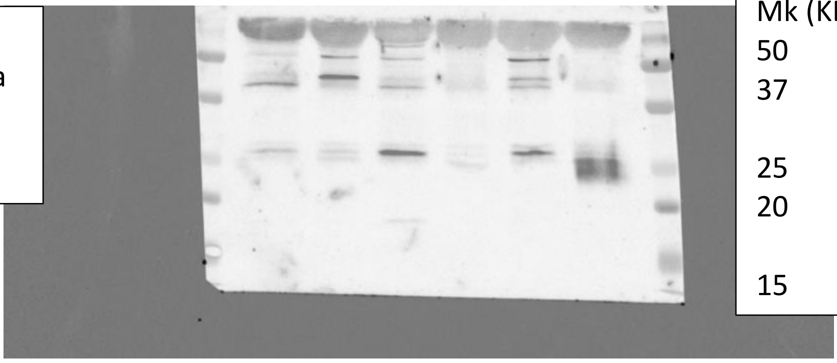

Mk (KDa)
50
37

25
20

15

X OXA C OXA C OXA

MT-COII – 22 kDa
NDUFB8 – 18 kDa

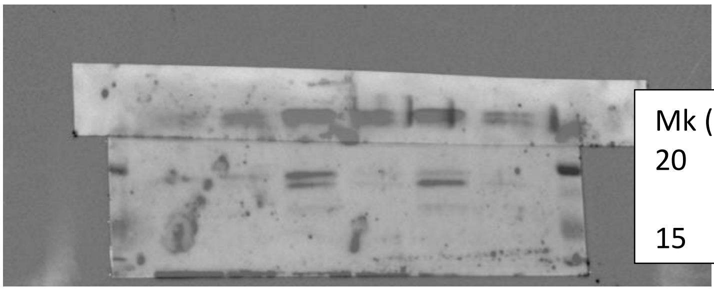

Mk (KDa)
20

15

X OXA C OXA C OXA

COXIV – 17 kDa

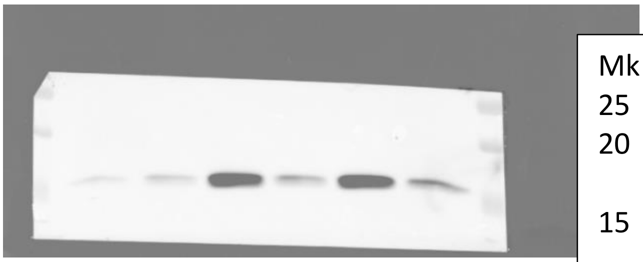

Mk (KDa)
25
20

15

X OXA C OXA C OXA

GAPDH – 37 kDa

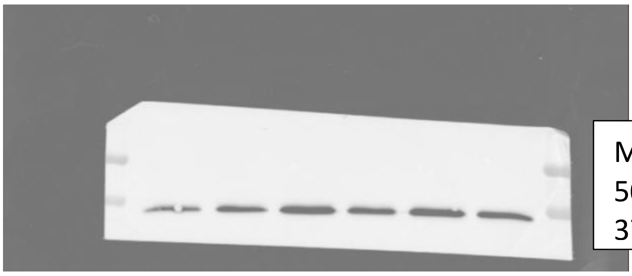

Mk (KDa)
50
37
